# Complete Genome Sequence of Shewanella sp. WE21, a Rare Isolate with Multiple Novel Large Genomic Islands

**DOI:** 10.1128/genomeA.00277-18

**Published:** 2018-04-19

**Authors:** Daniel Castillo, Lone Gram, Frank E. Dailey

**Affiliations:** a Marine Biological Section, University of Copenhagen, Helsingør, Denmark; b Department of Biotechnology and Biomedicine, Technical University of Denmark, Kongens Lyngby, Denmark; c Department of Biology, University of Wisconsin–Sheboygan, Sheboygan, Wisconsin, USA

## Abstract

We present here the whole-genome sequence of Shewanella sp. WE21, an unusual omega-3 fatty acid-producing bacterium isolated from the gastrointestinal tract of the freshwater fish Sander vitreus (walleye). This genome contains a number of unique, large genomic islands with genes not present in other Shewanella bacteria.

## GENOME ANNOUNCEMENT

Shewanella spp. are Gram-negative, motile bacilli that are found in many environments but most often in aquatic environments, particularly in marine ones. They are important for the turnover of organic material, and they are capable of dissimilatory reduction of various metals and other substances. (1). Also, several Shewanella strains have been reported as candidates for biotechnological applications such as omega-3 fatty acid production (2) and bioremediation of heavy metals (1). In contrast to their beneficial roles, Shewanella strains have been identified as major agents of fish spoilage (3) and recently as fish pathogens responsible for outbreaks of disease (4). Furthermore, they are increasingly being implicated as opportunistic human pathogens (5). Shewanella sp. WE21, isolated from the gastrointestinal tract of the freshwater fish species Sander vitreus (walleye) in Lac des Mille, Ontario, Canada (48°84'N, 90°51'W) (6), represents a novel omega-3 fatty acid-producing freshwater isolate of Shewanella. It appears most closely related to S. putrefaciens strains by phylogenetic analysis (6). However, *in silico* DNA-DNA hybridization suggests that Shewanella sp. WE21 is more closely related to (but distinct from) S. baltica species (F. Dailey, unpublished results). Here, we report the whole-genome sequence of this strain.

PacBio sequencing with 100× coverage was performed as previously described (6) for Shewanella sp. strain WE21. Annotation was performed by using the NCBI Prokaryotic Genome Annotation Pipeline (PGAP) (7). Additionally, the genomes were analyzed on the Rapid Annotations using Subsystems Technology (RAST) server (8). Acquired antibiotic resistance genes were identified by using ResFinder version 2.1 (9), virulence factors by using VirulenceFinder version 1.2 (10), clustered regularly interspaced short palindromic repeat (CRISPR) arrays by using CRISPRFinder (11), genomic islands by using IslandViewer version 4 (12), and prophage-related sequences by using PHASTER (13).

The final assembly for Shewanella sp. strain WE21 resulted in one large closed contig with a total length of 5,266,746 bp and a G+C content of 45.3%. Genome annotation resulted in 4,416 coding sequences (CDSs) plus one potential overlapping CDS, 105 tRNAs, 176 pseudogenes, and 31 rRNAs; 96.2% of the CDSs were classified as hypothetical proteins. Five prophage-like elements between 33.7 to 43.4 kb in length were detected. No virulence factors were identified in these prophage sequences. In addition, four genomic islands of 131.4 kb, 106.5 kb, 28.7 kb, and 9.2 kb, which have GC% contents of 40.6, 42.3, 38.3, and 57.9, respectively, were identified. In the first genomic island, we detected genes encoding putative virulence factors found in Vibrio anguillarum (14) and other fish pathogens, as well as a type III secretion system with large overlapping genes. Analysis of the second reveals not only additional genes encoding vibrio-like virulence factors, such as *cnf2*, but also genes encoding insecticidal toxins (15) and polyketides. The third genomic island putatively encodes very novel pili, and the fourth encodes copper efflux genes. Antibiotic resistance genes penicillin (*BL*) and fluoroquinolones (*parC*, *parE, gyrA*, and *gyrB*) were detected. Also, cobalt-zinc-cadmium resistance genes (*CzcD*, *CzcA*, *CusA*, *CusB*, and *TR*) and arsenic resistance genes were identified. No CRISPR arrays or plasmids were detected. This genome sequence can facilitate the understanding of symbiosis and virulence in fish intestinal bacteria.

### Accession number(s).

The draft genome sequence of Shewanella sp. strain WE21 can be accessed under the GenBank accession number CP023019. The version described in this paper is the first version.

## References

[1] Fredrickson JK, Romine MF, Beliaev AS, Auchtung JM, Driscoll ME, Gardner TS, Nealson KH, Osterman AL, Pinchuk G, Reed JL, Rodionov DA, Rodrigues JLM, Saffarini DA, Serres MH, Spormann AM, Zhulin IB, Tiedje JM 2008 Towards environmental systems biology of *Shewanella*. Nat Rev Microbiol 6:592–603. doi:10.1038/nrmicro1947. 18604222

[2] Amiri-Jami M, Griffiths MW 2010 Recombinant production of omega-3 fatty acids in *Escherichia coli* using a gene cluster isolated from *Shewanella baltica* MAC1. J Appl Microbiol 109:1897–1905. doi:10.1111/j.1365-2672.2010.04817.x. 20666868

[3] Vogel BF, Venkateswaran K, Satomi M, Gram L 2005 Identification of *Shewanella baltica* as the most important H2S-producing species during iced storage of Danish marine fish. Appl Environ Microbiol 71:6689–6697. doi:10.1128/AEM.71.11.6689-6697.2005. 16269698 PMC1287644

[4] Pękala A, Kozińska A, Paździor E, Głowacka H 2015 Phenotypical and genotypical characterization of *Shewanella putrefaciens* strains isolated from diseased freshwater fish. J Fish Dis 38:283–293. doi:10.1111/jfd.12231. 24552171

[5] Janda JM, Abbott SL 2014 The genus *Shewanella*: from the briny depths below to human pathogen. Crit Rev Microbiol 40:293–312. doi:10.3109/1040841X.2012.726209. 23043419

[6] Dailey FE, McGraw JE, Jensen BJ, Bishop SS, Lokken JP, Dorff KJ, Ripley MP, Munro JB 2016 The microbiota of freshwater fish and freshwater niches contain omega-3 fatty acid-producing *Shewanella* species. Appl Environ Microbiol 82:218–231. doi:10.1128/AEM.02266-15. 26497452 PMC4702627

[7] Tatusova T, DiCuccio M, Badretdin A, Chetvernin V, Nawrocki EP, Zaslavsky L, Lomsadze A, Pruitt KD, Borodovsky M, Ostell J 2016 NCBI Prokaryotic Genome Annotation Pipeline. Nucleic Acids Res 44:6614–6624. doi:10.1093/nar/gkw569. 27342282 PMC5001611

[8] Overbeek R, Olson R, Pusch GD, Olsen GJ, Davis JJ, Disz T, Edwards RA, Gerdes S, Parrello B, Shukla M, Vonstein V, Wattam AR, Xia F, Stevens R 2014 The SEED and the Rapid Annotation of microbial genomes using Subsystems Technology (RAST). Nucleic Acids Res 42:D206–D214. doi:10.1093/nar/gkt1226. 24293654 PMC3965101

[9] Zankari E, Hasman H, Cosentino S, Vestergaard M, Rasmussen S, Lund O, Aarestrup FM, Larsen MV 2012 Identification of acquired antimicrobial resistance genes. J Antimicrob Chemother 67:2640–2644. doi:10.1093/jac/dks261. 22782487 PMC3468078

[10] Joensen KG, Scheutz F, Lund O, Hasman H, Kaas RS, Nielsen EM, Aarestrup FM 2014 Real-time whole-genome sequencing for routine typing, surveillance, and outbreak detection of verotoxigenic *Escherichia coli*. J Clin Microbiol 52:1501–1510. doi:10.1128/JCM.03617-13. 24574290 PMC3993690

[11] Grissa I, Vergnaud G, Pourcel C 2007 CRISPRFinder: a web tool to identify clustered regularly interspaced short palindromic repeats. Nucleic Acids Res 35:W52–W57. doi:10.1093/nar/gkm360. 17537822 PMC1933234

[12] Langille MG, Brinkman FS 2009 IslandViewer: an integrated interface for computational identification and visualization of genomic islands. Bioinformatics 25:664–665. doi:10.1093/bioinformatics/btp030. 19151094 PMC2647836

[13] Arndt D, Grant J, Marcu A, Sajed T, Pon A, Liang Y, Wishart DS 2016 PHASTER: a better, faster version of the PHAST phage search tool. Nucleic Acids Res 44:W16–W21. doi:10.1093/nar/gkw387. 27141966 PMC4987931

[14] Castillo D, Alvise PD, Xu R, Zhang F, Middelboe M, Gram L 2017 Comparative genome analyses of *Vibrio anguillarum* strains reveal a link with pathogenicity traits. mSystems 2:e00001-17. doi:10.1128/mSystems.00001-17. 28293680 PMC5347184

[15] Bowen D, Rocheleau TA, Blackburn M, Andreev O, Golubeva E, Bhartia R, Ffrench-Constant RH 1998 Insecticidal toxins from the bacterium *Photorhabdus luminescens*. Science 280:2129–2132. doi:10.1126/science.280.5372.2129. 9641921

